# Implication of food insecurity on the gut microbiota and its potential relevance to a multi‐ethnic population in Malaysia

**DOI:** 10.1002/jgh3.12709

**Published:** 2022-02-01

**Authors:** Nor H Shafiee, Nurul H Razalli, Khairul N Muhammad Nawawi, Norfilza Mohd Mokhtar, Raja Affendi Raja Ali

**Affiliations:** ^1^ Department of Medicine, Faculty of Medicine Universiti Kebangsaan Malaysia Kuala Lumpur Malaysia; ^2^ Dietetics Programme, Faculty of Health Sciences Universiti Kebangsaan Malaysia Kuala Lumpur Malaysia; ^3^ GUT Research Group, Faculty of Medicine Universiti Kebangsaan Malaysia Kuala Lumpur Malaysia; ^4^ Gastroenterology Unit, Department of Medicine UKM Medical Centre Kuala Lumpur Malaysia; ^5^ Department of Physiology, Faculty of Medicine Universiti Kebangsaan Malaysia Medical Centre Kuala Lumpur Malaysia

**Keywords:** diet, food insecurity, gut microbiota, multi‐ethnic population, nutrition

## Abstract

Food insecurity (FI) has an impact on food intake, and it can make it difficult for people to eat enough nutritious food at all times to sustain an active and healthy lifestyle. The COVID‐19 outbreak has hampered people's capacity to obtain nutritious and affordable food. Although FI has been studied in Malaysia, the extent to which it is linked to gut microbiota has yet to be discovered. This review aimed to compile evidence of the relationship between FI and gut microbial changes and their potential relevance to a multi‐ethnic population in Malaysia. FI is typically associated with cheaper and calorie‐dense foods because of the high cost of quality food and financial constraints that hinder food‐insecure people from adopting healthier dietary choices. As a result, they have started eating low‐quality food such as simple carbohydrates, fats, and processed foods. These poor eating habits can reduce microbial diversity and influence changes in the composition and function of the gut microbiota. This review also explores the impact of ethnicity on the variation in composition of gut microbiota. In conclusion, the findings of this review may be utilized to develop and implement diet‐related intervention programs to ensure that Malaysians get enough nutritious food to maintain a healthy gut microbiota and improve overall health.

## Graphical abstract

Food insecurity refers to lack of access to a sufficient quantity of nutritious diets, which may influence changes in gut microbiota composition. The Covid‐19 pandemic has exacerbated the existing food insecurity in Malaysia. Thus, nutrition‐related intervention programs that focus on healthy food choices are important to establish and maintain a healthy gut community and overall health.

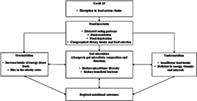

## Introduction

**Figure 1:**
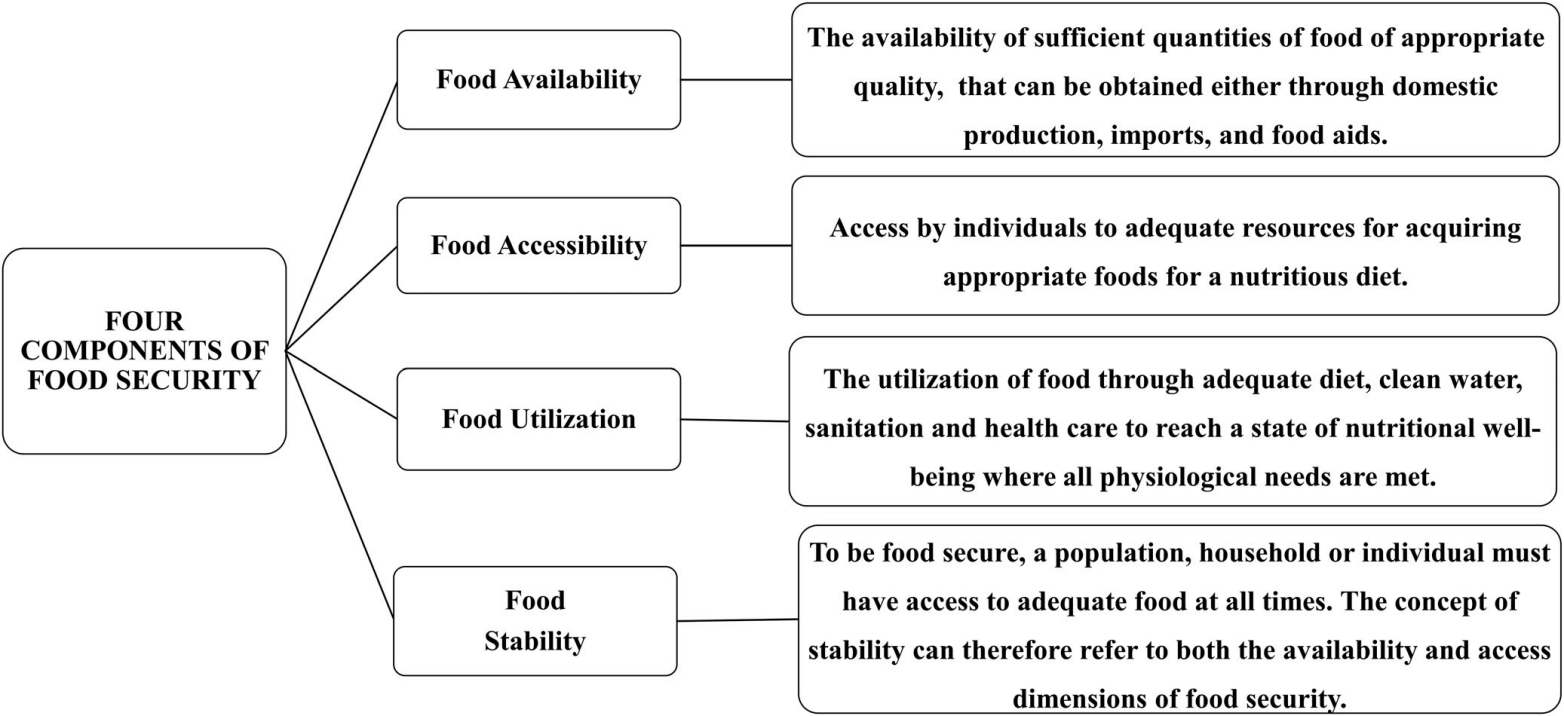
The four components of food security.

Food insecurity (FI) is a major nutritional issue that affects people all over the world. It is defined as a situation in which people have limited physical, social, and economic access to enough safe and nutritious food to meet their dietary needs and food preferences to sustain an active and healthy life.^1^ In general, FI is measured along four dimensions, as shown in Figure 1: (i) food availability; (ii) access to sufficient quantities and a diverse range of safe, high‐quality food; (iii) food utilization; and (iv) food supply stability.^2^ It can occur at any level, from the individual to the household, community, regional, national, and global,^3^ and everyone is accountable for ensuring food security. For example, food security at the household level refers to a household's and all of its members' ability to acquire enough food to meet their dietary needs, regardless of the sources of food, which can include food production or purchase.^4^

FI is a risk factor for all types of malnutrition, including deficiency, excess or imbalance of energy, and other macro and micronutrients, as well as undernutrition and overnutrition (overweight or obesity). Because of insufficient intake and overconsumption of high‐calorie/low‐nutrient‐dense foods,^1^, ^5^ FI can lead to a reduction in dietary quality, resulting in poor nutrition.^6^ Low income, poverty, inadequate dietary intake, and poor nutritional status are all linked to household food insecurity (HFI).^7^, ^8^ Some studies have found that FI is prevalent in households with poor socioeconomic levels in both rural and urban parts of Malaysia. According to a study conducted in rural sub‐districts of Bachok, Kelantan, the prevalence of FI in low‐income households was greater (83.9%)^9^ than in earlier studies.^10^, ^11^ Meanwhile, another survey found that 73.8% of urban welfare‐recipient households in Hulu Langat, Selangor, were food insecure.^12^

As a result of the government's Movement Control Orders (MCO) strategy to reduce the disease's infection rate in the country, the emergence of coronavirus disease (COVID‐19) could exacerbate the existing FI in Malaysia. Food production, transportation, and services have all been impacted by MCO or “lockdown restriction” enforcement, posing a significant challenge to the food system's ability to supply enough affordable and healthy food for everyone.^13^ The negative impacts of COVID‐19 and MCO on jobs and livelihoods also contribute to the risk of FI. Individuals who are experiencing a reduction in income or job losses may be concerned about developing poor eating habits,^14^ which may limit their ability to purchase or prepare healthy meals for themselves and their families. Furthermore, according to the Malaysian Dietary Guidelines (MDG), the cost of cooking nutritious food for a balanced diet at home is between MYR 756.30 (USD 178.79) and MYR 1153.50 per month (USD 272.70), which is around the poverty line income (PLI) of MYR 980 (USD 243).^15^ As a result, impoverished households, particularly those in the B40 category (the bottom 40% of income earners), may have to carefully budget their whole monthly income to purchase sufficient healthy food daily.

In Malaysia, the pandemic impacts not only the B40 groups but also the M40 groups (the middle 40% of income earners), whose food patterns may be directly distorted by salary cuts and job losses, putting them at risk of FI. According to a survey by Ismail *et al*., 35.4% of M40 respondents have been laid off, got only half‐pay, been placed on unpaid leave, or shuttered their business as a result of the MCO.^16^ Furthermore, the majority of M40 respondents (41.5%) had only 1–3 months' worth of savings, and if MCO is extended for a longer term, it will alter the respondents' consumption and expenditure patterns, including food supply resistance and buying patterns.^16^ As a result, the distorted food supply will disrupt food security and have an impact on people's nutritional intake,^15^, ^17^ as they may limit the amount of food they consume and eat less frequently than usual.

During the current pandemic, FI may lead to households consuming low‐cost, calorie‐dense, nutrient‐poor meals, which may have an impact on the gut microbiota. High consumption of cereals, fruit, vegetables, and legumes has been shown to have a positive impact on the composition of the gut microbiota.^18^ Meanwhile, a highly refined carbohydrate diet can lead to dysbiosis.^19^ As previously stated, FI is linked to poor dietary quality, which leads to nutritional inadequacies. According to a study, those who are food‐insecure consume fewer vegetables, fruits, and dairy products, and have lower intakes of vitamins A and B6, calcium, zinc, and magnesium than those who are food‐secure.^20^ While there has been some research on FI in Malaysia, the extent to which it is linked to the gut microbiota has yet to be determined. As a result, the focus of this review is on the relationship between FI and gut microbial changes and their potential relevance to a multi‐ethnic population in Malaysia.

## Methods

### Search strategy and study selection

A fullly automated literature search approach was employed to discover relevant papers for this review, using electronic database search engines such as PubMed, Scopus, EMBASE, and Google Scholar. Free text words and medical subject heading (MeSH) phrases were utilized to improve sensitivity and refine the search. The terms “food insecurity,” “FI,” “gut microbiota,” “microbiome,” “nutritional status,” “diet,” and “gut dysbiosis” were used to conduct the literature search. The Boolean operators (AND, OR) were used to narrow or broaden the search results. The search was limited to English‐language publications. The research covered a wide range of study designs from 2010 to 2021, including cross‐sectional studies, cohort studies, interventional studies, epidemiological studies, and randomized control studies (RCTs). Human and animal studies were also taken into consideration.

## Food insecurity's consequences and nutritional outcomes in the Malaysian population

Through impaired diets, FI can have a negative impact on a person's nutritional state, as seen by insufficient food consumption, a lack of dietary diversity, and poor diet quality. These conditions may lead to a reduction in caloric and nutritional intake, especially vitamins and minerals, resulting in a high prevalence of overweight, obesity, or at‐risk waist circumference (WC). Several groups of Malaysians, including migrant workers, indigenous peoples, low‐income households, adolescents, university students, and the elderly, were recognized to be at a higher risk of FI before the emergence of COVID‐19 (Table 1).

**Table 1:** Summary of studies reviewing food insecurity and nutritional outcomes in Malaysia

Respondents (target groups)	Setting	Study design	Food insecurity assessment tool	Nutritional outcomes measured	References
Migrant workers (*n* = 125)	Klang Valley, Selangor	Cross‐sectional	Household Food Insecurity Access Scale (nine items)	Nutrients intake, weight, height, body mass index (BMI), and waist circumference (WC)	21
Indigenous women (*n* = 222)	Carey Island and Tanjung Sepat, Kuala Langat, Selangor	Cross‐sectional	The Radimer/Cornell Hunger and Food Insecurity Instrument (10 items)	Food intake, diet quality, height, weight, BMI	23
Indigenous women (*n* = 92)	Gombak, Selangor	Cross‐sectional	The Radimer/Cornell Hunger and Food Insecurity Instrument (10 items)	Dietary intake, weight, height, WC, BMI	26
Low‐income households (*n* = 169)	Palm plantation, Negeri Sembilan	Cross‐sectional	Radimer/Cornell Hunger and Food Insecurity Instrument (10 items)	Nutrient intakes, diet diversity, number of servings from each food groups, weight, height, WC, and BMI	24
Low‐income households (*n* = 625)	Selangor, Negeri Sembilan, Kelantan,	Cross‐sectional	Radimer/Cornell Hunger and Food Insecurity Instrument (10 items)	Diet diversity, weight, height, BMI, and WC	27
Low‐income households (*n* = 223)	Bachok, Kelantan	Cross‐sectional	Radimer/Cornell Hunger and Food Insecurity Instrument (10 items)	Weight, height, BMI, and WC	28
Adolescents (*n* = 160)	Mentakab, Pahang,	Cross‐sectional	Radimer/Cornell Hunger and Food Insecurity Instrument (10 items)	Weight, height, and BMI	29
Elderly (*n* = 289)	Felda Land Development Authority (FELDA) Lubuk Merbau, Kedah, and Northern Region of Malaysia	Cross‐sectional	Food Security Tool for Elderly	BMI	31
Elderly (*n* = 72)	Klang Valley, Selangor	Cross‐sectional	Radimer/Cornell Hunger and Food Insecurity Instrument (10 items)	BMI and WC	32
University students (*n* = 108)	Public University, Selangor	Cross‐sectional	USDA Six‐item Short Form of Survey Module	BMI and nutrients intake	36

According to a survey of documented migrant workers in Malaysia's Klang Valley, 57.6% of the households were food‐insecure, with low mean calorie intakes, and most of the nutrients (carbohydrate, protein, fat, vitamin C, calcium, iron, and dietary fiber) were below the recommended levels.^21^ These findings could be due to a rigorous work schedule that left them with little time to prepare nutritious and safe meals and to eat adequate amounts of food. With the current pandemic, the situation may deteriorate further, as many workers, especially migrant workers, are unable to work, resulting in a significant loss of income,^22^ limiting access to better food in terms of type, variety, and quantity, as they did before the MCO was implemented.

Despite being a minority, the indigenous people in Malaysia have been observed to have a high prevalence rate of FI. A survey of indigenous women in Peninsular Malaysia revealed that 82.9% of households had FI, with the food‐insecure group having a lower diet quality, *P* = 0.023, as evidenced by a lower intake of grains and cereals, as well as meat, poultry, and eggs, compared to their food‐secure counterparts,^23^ which is consistent with previous local studies.^19^, ^24^, ^25^ These findings could be attributed to a lack of financial access to a variety of dietary options, as well as an increase in food prices, which limit their ability to obtain nutritious foods in sufficient quantities. As Malaysia continues to implement lockdown measures in response to an increase in COVID‐19 cases, poor and rural indigenous people who already face FI will have even more difficulty in obtaining food. However, individuals may be able to cope and find a way to lessen the impact of FI by eating whatever food is available around the house (66.3%), such as plant‐based foods, and purchasing less expensive food (64.1%).^26^ In addition, rural indigenous people in Malaysia ate a lot of wild vegetables such as tapioca shoots and swamp cabbage, as well as non‐seasonal fruits such as banana and papaya, which were either cultivated locally or picked in the forest,^27^ and could help them acquire more fiber.

FI is indirectly linked to poor nutritional status. According to a study in an oil palm plantation household in Negeri Sembilan, Malaysia, food‐insecure women had a lower mean intake of vitamin A, diet diversity score (DDS), and the number of servings from meat, fish, poultry, and legumes than food‐secure women, *P* < 0.05.^24^ In addition, that study found a high prevalence of at‐risk abdominal obesity (WC ≥88 cm) (68.8%) and a significant relationship with FI (*P* < 0.05).^24^ These findings could be explained by their low income, which has led them to adopt coping strategies such as eating a less diverse diet and favoring cheaper, energy‐dense foods to boost their energy consumption. As Zalilah *et al*. discovered, when compared to food‐secure women, women in food‐insecure households were less likely to have abdominal obesity, metabolic syndromes (MetS), high glucose, and low‐density lipoprotein cholesterol (LDL‐c), *P* < 0.05.^28^ However, their study did not assess the sufficiency of energy and nutrient intake or food group servings to determine whether HFI increases the risk of nutritional deficiencies in individuals.

Despite the high incidence of overweight and obese women (52%), with 47.1% having at‐risk WC (≥80 cm), another study found no significant link between FI, body mass index (BMI), *P* = 0.896, and abdominal obesity, *P* = 0.438.^29^ Because obesity is a chronic illness that develops over months or years, these findings could be explained by the fact that FI experienced by households at a single point in time was not significantly or consistently related to women's subsequent weight increase and obesity. In contrast, a more recent study in the rural region of Mentakab, Pahang, indicated that food‐insecure adolescents had a significantly lower BMI than their food‐secure counterparts, *P* < 0.001.^30^ Food‐insecure households are more likely to eat smaller amounts of meals, which contributes to their lower overall energy consumption. Meanwhile, the Malaysian Health and Adolescents Longitudinal Research Team Study (MyHeARTs Study) found that rural adolescents consume more calories and cholesterol than their urban counterparts,^31^ possibly due to a higher intake of calorie‐dense foods.

During the COVID‐19 outbreak, the prevalence of FI levels among the elderly population was lower (14.8%)^32^ than in a previous study, which found that 27.7% of older respondents had FI.^33^ According to this study, a potential explanation for this outcome could be that most of the respondents were living with their families during the MCO and that, since they were working from home, the children were able to provide adequate and nutritious meals for their parents.^32^ In a previous study, Rohida *et al*. found that 51.6% of senior adults were overweight or obese,^33^ while another study found that 75.0% of elderly people had abdominal obesity.^34^ According to Zainuddin *et al*., fat consumption was also linked to FI (*r* = −0.280, *P* = 0.017), which could lead to an increased risk of fat‐frailty.^34^ Higher BMI and waist‐to‐hip ratio (WHR) among the respondents could be linked to lifestyle and physiological changes that contribute to fat accumulation due to a decrease in physical activity, as well as an increase in age‐related diseases and functional decline.^35^

University students were also a particularly vulnerable group when it came to FI. According to published surveys, the incidence of FI among university/college students in Malaysia ranged from 22.0 to 69.4%.^36^, ^37^, ^38^, ^39^, ^40^, ^41^ The primary reasons leading to FI among students have been identified as an increase in tuition expenses, insufficient student financial help, and high living costs.^36^, ^39^ Most Malaysian university students adopt poor eating habits to cope with FI, which includes irregular meals, the consumption of unhealthy foods such as instant noodles or fast food, and a low intake of fruits and vegetables.^39^, ^40^ In terms of nutritional consumption, Norhasmah *et al*. found that nearly all of the university students (96.7%) had a poor intake of folate, calcium, vitamin C, vitamin A, and thiamine when compared to the Malaysian recommended intake.^39^ Meanwhile, Rajikan *et al*. discovered that most university students had lower calorie and protein intakes, but greater fat intakes, when compared to their nutritional needs.^37^ In their study, however, no significant relationship was found between energy and macronutrient consumption and food security status (*P* = 0.68).^37^ This was most likely due to the restricted variety of food available in the university cafeteria, which resulted in no statistically significant differences in the eating patterns of all students.^37^

## Impact of food insecurity on the gut microbiota

In 2020, Christian *et al*. examined studies on the effects of FI on malnutrition and the gut microbiota.^42^ They found that when people go without meals, their gut flora changes.^42^ The authors also emphasized that gut microbiota dysbiosis and immaturity due to FI were linked to malnutrition during the current pandemic. As a result, Littlejohn *et al*. pointed out in a recent opinion paper that changes in the gut microbiota can be significantly influenced by both undernutrition and obesity in times of the COVID‐19 epidemic, with altered gut community structure and function being similar in both.^43^ Furthermore, COVID‐19 infection in combination with FI was found to promote gut microbiota dysbiosis, which could have acute or long‐term health implications.^43^

The composition and function of the gut microbiota in a state of dietary restriction and deprivation were also disclosed in a review by Genton *et al*.^44^ This was supported by Monira *et al*., who found that malnourished children had a significantly higher fecal proportion of *Proteobacteria* and a lower fecal proportion of *Bacteroidetes* when compared to their healthy counterparts.^45^ Healthy children in this study were from households with moderate to high socioeconomic status, while malnourished children came from low‐income families. The microbial diversity was significantly lower in the malnourished children, with the abundance of pathogenic *Proteobacteria* being 9.2 times higher than in healthy children.^45^ Despite the lack of studies on the influence of FI on the gut microbiome, a recent pilot study in 3‐month‐old infants (*n* = 68) found that those from food‐insecure households had less microbial diversity than those from secure families.^46^ More research is needed in the future to validate these findings.

**Figure 2:**
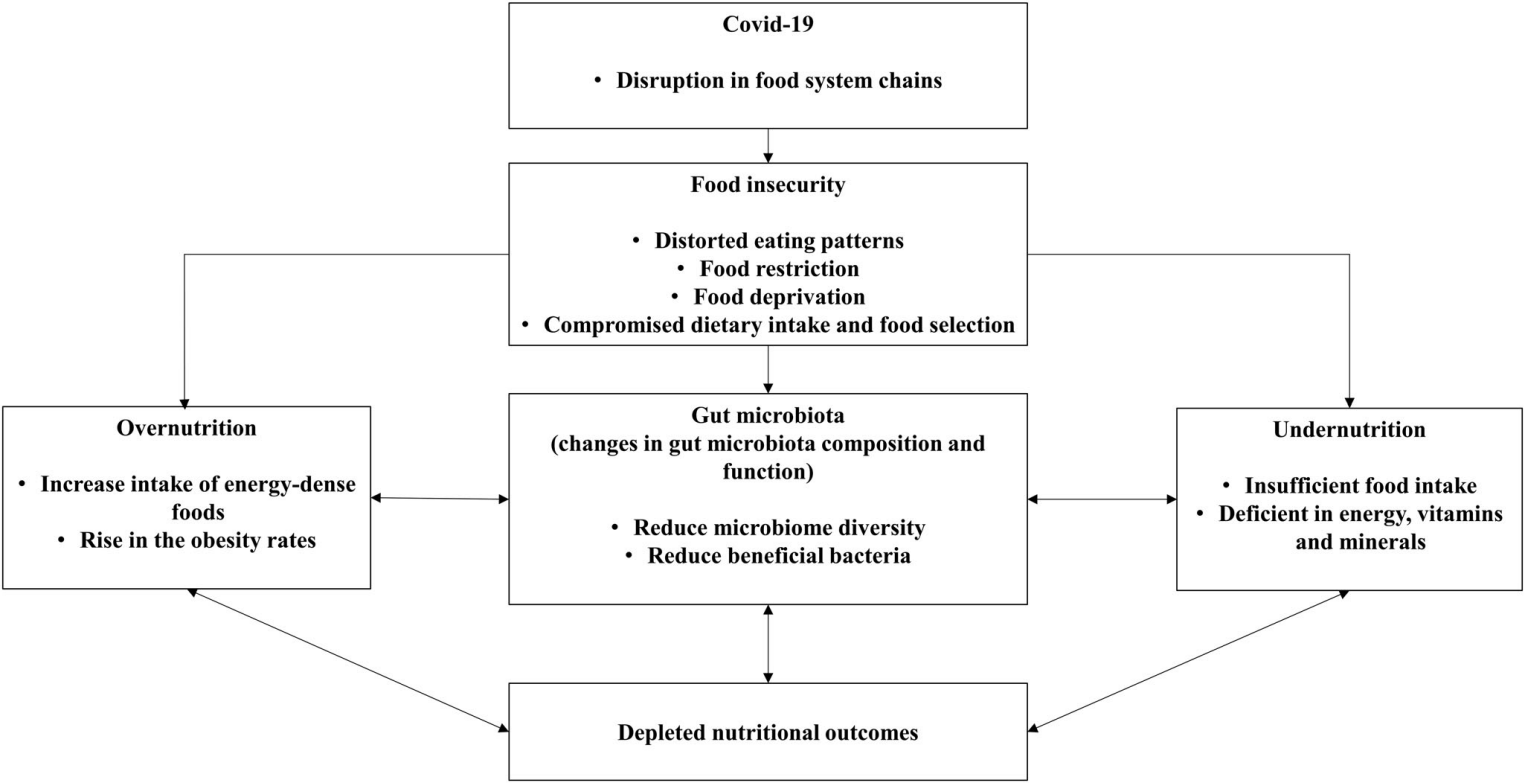
Potential mechanism interlinked food insecurity, malnutrition, and gut microbial changes.

In this review, we describe our understanding of the potential mechanism underlying the link between FI and changes in gut microbiota in a state of malnutrition (Fig. 2). The types and frequency of food consumed may also have an impact on the composition and function of the gut microbiota as well as bacterial diversity. As a result, we briefly discuss several unhealthy dietary habits related to FI, including the consumption of high‐fat foods, a higher intake of simple carbohydrates, and processed foods.

### Consumption of diets rich in fat

Bisanz *et al*. conducted a meta‐analysis using sequencing data from 25 studies on the impact of a high‐fat diet (HFD) on gut microbiota composition in animals and humans.^47^ In most trials, the HFD increased the ratio of *Firmicutes* to *Bacteroidetes*. Furthermore, when compared to the low‐fat diet (LFD) group, the HFD group showed significant changes in microbial composition, indicating that HFD can alter gut microbiome composition. Other studies have found that HFD can change the gut microbiota composition regardless of the duration of the feedings.^48^, ^49^ David *et al*. reported a rapid shift in gut microbiome structure and function after only 2 days of HFD consumption, as evidenced by an increase in the number of bile‐tolerant bacteria (*Alistipes* spp., *Bacteroides* spp., and *Bilophila* spp.) and a decrease in *Firmicutes*.^48^ Meanwhile, a cross‐sectional study found that long‐term HFD consumption, rather than short‐term dietary alterations, can lead to an increase in the *Bacteroides* genus.^49^

### Consumption of refined carbohydrates

The deleterious effects of added sugars, especially sugar‐sweetened beverages (SSBs), have been linked to an unfavorable gut microbial composition. The largest epidemiological study of Swedish adults found that SSB consumption was linked to a higher *Firmicutes*: *Bacteroidetes* ratio and a lower percentage of beneficial butyrate‐producing bacteria (*Lachnobacterium*).^50^ Furthermore, in contrast to a previous observational study, which found a negative relationship between fructose intake and the abundance of *Streptococcus* and *Eubacterium*,^51^ this study found a positive relationship between added sugar intake and the *Streptococcus* genera and positive relationships between SSB and artificially sweetened beverage (ASB) intake and *Eubacterium*.^50^

### Consumption of processed foods

As a result of MCO, people will consume more processed foods such as snacks, ready‐to‐eat cereal, and fast food, which are high in fat, sugar, and salt.^52^ An experimental study in C57BL/6 mice found that eating processed foods increased the *Firmicutes* to *Bacteroidetes* ratio, driven mainly by the reduction in the *Bacteroidia* class as well as an increase in the abundance of *Enterobacteriaceae* and *Verrucomicrobia*.^53^ Bolte *et al*. also found that a high dietary intake of processed foods was consistently associated with a higher abundance of *Firmicutes* and *Ruminococcus* spp. of the *Blautia* genus.^54^

## Ethnic differences in gut microbiota and food insecurity

Malaysia is a multi‐ethnic country with a population of 32.7 million people in 2021.^55^ It is made up of four primary ethnic groups: Malay, Chinese, Indian, and a minority of indigenous people. A few studies in Malaysia have produced conflicting results regarding the impact of ethnicity on the gut microbiota.^56^, ^57^, ^58^ A better understanding of how gut microbiota distribution varies across ethnicities could help speed up the development of strategies to reduce the risk of gut‐related diseases.

In a rural location in Northern Malaysia, Chong and colleagues discovered that ethnicity and socioeconomic status had a significant impact on gut microbiota diversity in pre‐adolescents from three ethnic groups (Malays, Chinese, and Orang Asli [indigenous]).^56^ In comparison to children of wealthy Chinese and Malays, indigenous children who are comparatively economically challenged exhibited the most microbial diversity.^56^ Furthermore, based on 16S rDNA‐based pyrosequencing, *Aeromonadales*, *Ruminococcaceae*, *Bacteroidetes*, *Deltaproteobacteria*, and *Spirochaetes* were enriched in the former gut microbial profiles, which have been linked to the breakdown of fiber‐rich foods such as *ulam* (traditional salad), which is widely consumed by the indigenous community.^59^

In a comparison of pre‐adolescents from Guangzhou City (China), Penang City (west coast Malaysia), and Kelantan City (Malaysia), a recent study by Khine *et al*. found that food habits, rather than ethnicity alone, were a key driver of microbiome alterations (east coast Malaysia).^57^ The findings indicated that the differences in gut microbiota composition among ethnicities in various cities were caused by changes in the sorts of foods ingested.^57^ *Bifidobacterium* and *Collinsella* were prevalent in the gut microbiota of Chinese people in two urbanized Malaysian locations, and both were positively associated with refined sugar‐enriched diets.^57^ *Collinsella* was also favorably associated with fruits and curry dishes but negatively associated with Southeast Asian veggies, whereas *Bacteroides*, *Fecalibacterium*, and *Bifidobacterium* were adversely associated with high‐fat meals.^57^

Another study by Dwiyanto *et al*. looked at the impact of ethnicity on the gut microbiota of adults in a multi‐ethnic population (Malay, Chinese, Indian, and indigenous people) from a single area in Malaysia's southern peninsula.^58^ Their findings showed that lifestyle and nutritional variables influenced gut microbiome diversity among ethnic groups in the same community, making ethnicity a reasonable proxy for these aspects across ethnic groups within the same community.^58^ After adjusting for all other variables such as demographics, dietary behavior, hygiene practices, and health conditions, the results revealed that ethnicity had the greatest impact on the gut microbiota (permanova Pseudo‐*F* = 1.67, *R* ^2^ = 0.02, *P* = 0.002).^58^

## Recommendations

With the available information in this review, quick action or targeted intervention programs are needed to minimize the prevalence of FI among Malaysia's vulnerable populations, particularly during this COVID‐19 pandemic. Given FI and its effects, the government must implement the existing National Plan of Action for Nutrition of Malaysia III (NPANM III), 2016–2025, to promote food security and enhance Malaysians' nutritional status.^60^ NPANM III required the collaboration of various ministries and government agencies, research institutions, academia, professional bodies, nongovernmental organizations (NGOs), and the private sector, such as the food industry, to promote safe and diversified healthy eating by increasing food quality and diversity, increasing purchasing power, and reducing unhealthy eating behaviors.^60^

Both the federal and state governments have implemented various incentives to mitigate the disruption and losses caused by the pandemic crisis by expanding financial help, including food‐based support such as Food Bank Malaysia and Food Aid Packages. Furthermore, the Malaysian government, in collaboration with the business sector, the media, and NGOs, should work to enhance the functioning of food supply chains so that everyone has access to a variety of healthy meals and their food security is improved. All related sectors should collaborate to improve the nutritional quality of food produced and available on the market, promote the marketing of diverse and nutritious foods, and lower the cost of nutritious foods to ensure that people can obtain adequate quantities and quality of food, as well as improve the nutritional status of poor households in the country.

## Conclusion

In conclusion, this review should assist professionals in recognizing FI as one of the variables that negatively affect alterations in the gut microbiome, particularly in terms of food quality, quantity, and variety. The findings showed that FI was linked to low nutritional status, especially among Malaysia's poor and vulnerable. The significance of dietary‐related FI in reshaping the structure of the gut microbiota and altering the composition, functioning, and changes in biodiversity via modifying metabolite synthesis is highlighted in this study. Researchers, health practitioners, nutritionists, and policymakers can use the information from this review to devise nutritional coping strategies that emphasize the availability and accessibility of healthy food options to establish and maintain a healthy gut community and improve overall health. Malaysians' adoption of healthy eating habits is still low, which is due to the high cost of nutritious foods. However, with government and business support on food costs, Malaysians' acceptance of healthy food intake may increase. Furthermore, nutrition education programs may improve Malaysians' understanding of good eating practices.

## Future research

This review could be further expanded in the future to investigate and comprehend the food security concerns that have arisen as a result of the current COVID‐19 pandemic, as well as the effects on gut microbiota composition. Furthermore, future research should continue to focus on potential diet‐related treatments for managing FI‐related gut dysbiosis, which could include nutrition education as a means of increasing access and availability to healthier foods, particularly among the poor and vulnerable.
